# Quercetin Enhances Inhibitory Synaptic Inputs and Reduces Excitatory Synaptic Inputs to OFF- and ON-Type Retinal Ganglion Cells in a Chronic Glaucoma Rat Model

**DOI:** 10.3389/fnins.2019.00672

**Published:** 2019-06-25

**Authors:** Xujiao Zhou, Gang Li, Boqi Yang, Jihong Wu

**Affiliations:** ^1^Eye Institute, Eye and ENT Hospital, State Key Laboratory of Medical Neurobiology, Institutes of Brain Science and Collaborative Innovation Center for Brain Science, Shanghai Medical College, Fudan University, Shanghai, China; ^2^Shanghai Key Laboratory of Visual Impairment and Restoration, Shanghai, China; ^3^NHC Key Laboratory of Myopia, Key Laboratory of Myopia, Chinese Academy of Medical Sciences, Fudan University, Shanghai, China; ^4^Department of Ophthalmology and Vision Science, Eye and ENT Hospital, Fudan University, Shanghai, China

**Keywords:** quercetin, inhibitory synaptic inputs, excitatory synaptic inputs, retinal ganglion cells, glaucoma, patch-clamp

## Abstract

**Background:**

Glaucoma is a neurodegenerative disease caused by excitotoxic injury of retinal ganglion cells (RGCs). In our previous model of high intraocular pressure, prepared by injecting magnetic beads into the anterior chamber, we demonstrated that an important natural dietary flavonoid compound (quercetin) can improve RGC function. However, it is unclear whether quercetin can improve the synaptic function of RGCs and how quercetin regulates synaptic transmission in rat models of chronic glaucoma.

**Methods:**

A rat model of chronic glaucoma was prepared by electrocoagulation of the superior scleral vein. Electrophysiological electroretinography was used to detect the photopic negative response (PhNR). The whole-cell patch-clamp technique was used to clamp ON- and OFF- type RGCs in sections from normal retinas and from retinas that had been subjected to glaucoma for 4 weeks.

**Results:**

Quercetin can reverse the decrease in PhNR amplitude caused by chronic glaucoma. The baseline frequency of miniature GABAergic inhibitory postsynaptic currents (mIPSCs) in the RGCs of glaucomatous retinal slices was lower than that of the control group. The frequencies of miniature excitatory postsynaptic currents (mEPSCs) were not significantly different between control and glaucomatous RGCs. The baseline frequencies of GABAergic mIPSCs and mEPSCs in OFF-type glaucomatous RGCs were greater than those in ON-type glaucomatous RGCs. Quercetin increased miniature GABAergic inhibitory neurotransmission to RGCs and decreased miniature glutamatergic excitatory neurotransmission, reducing the excitability of the RGCs themselves, thus alleviating the excitability of RGCs in glaucomatous slices.

**Conclusion:**

Quercetin may be a promising therapeutic agent for improving RGC survival and function in glaucomatous neurodegeneration. Quercetin exerted direct protective effects on RGCs by increasing inhibitory neurotransmission and decreasing excitatory neurotransmission to RGCs, thus reducing excitotoxic damage to those cells in glaucoma.

## Introduction

Quercetin is the most abundant flavonoid and is found in fruits and vegetables such as onions, broccoli, apples and berries (Mukhtar, 1996; Miean and Mohamed, 2001). Quercetin can penetrate the blood-brain barrier (Youdim et al., 2004a,b) to exert anti-inflammatory, antihistamine and antioxidant effects (Ross and Kasum, 2002; Tang et al., 2005). Quercetin can alleviate neurotoxic injury induced by peroxidation (Ishige et al., 2001), oxygen and glucose deprivation (Ha et al., 2003) or neurotoxicity (Ansari et al., 2009). Previous studies in our laboratory and other laboratories have shown that quercetin has neuroprotective effects. Our research group (Gao et al., 2017) found that quercetin not only improves retinal ganglion cell (RGC) survival and function from a very early stage in chronic ocular hypertension *in vivo* but also promotes the survival of hypoxia-treated primary cultured RGCs *in vitro* by improving mitochondrial function and preventing mitochondria-mediated apoptosis.

The firing state of neurons depends on a dynamic balance between excitability and inhibitory synaptic afferents, and disruption of this circuit can lead to neurodegenerative diseases, such as glaucoma (Moreno et al., 2008; Nelson and Valakh, 2015). Impairments in the excitability/inhibitory balance involve a variety of potential pathologic mechanisms, such as impaired inhibitory interneurons, the cumulative excitotoxicity of glutamate, and the abnormal expression and function of presynaptic and postsynaptic protein molecules (Moreno et al., 2008; Sudhof, 2008; Nakazawa et al., 2012). Therefore, restoring the excitation-inhibition balance might be a promising novel therapeutic strategy.

The effects of quercetin and its related compounds on the ionic current of neurons in the central nervous system have been widely studied. The electrophysiological results showed that quercetin can regulate many ligand-gated ion channels, including GABAA and GABAC receptors, glycinergic receptors, glutamate kainate receptors, 5-HT receptors and nicotinic acetylcholine receptors (Lee et al., 2002; Goutman et al., 2003). However, the mechanism by which quercetin acts on RGC synaptic transmission in the rat retina has not been studied. An answer to this open question may clarify the regulatory mechanism of quercetin at the presynaptic level in the whole neural network. Therefore, we prepared an animal model of chronic glaucoma and used this model to study the mechanism of quercetin’s neuroprotective effect at the presynaptic afferent level. As an important excitatory neurotransmitter of the central nervous system (CNS), glutamate plays an important role in synaptic plasticity, learning and memory (Greenamyre and Porter, 1994; Lu et al., 2013). In addition to physiological functions, glutamate in synaptic clefts has potential excitotoxicity after excessive release. Excessive glutamate release is one of the molecular mechanisms responsible for many neuropathic neuron injuries, including acute traumatic stroke, seizures, and traumatic brain and spinal cord injury, and chronic neurodegenerative diseases such as Parkinson’s disease and Alzheimer’s disease (Lu et al., 2013). Therefore, effective reduction of glutamate release would be considered a potential neuroprotective strategy. In fact, some neuroprotectants currently used in human and animal brain tissue act by reducing glutamate release (Wang et al., 2004). Wang et al. (2004), Lu et al. (2013) provided evidence that quercetin inhibits glutamate release from purified rat cerebrocortical synaptosomes with a fluorometric assay that used exogenous glutamate dehydrogenase (GDH) and NADP^+^ to couple the oxidative deamination of released glutamate to the generation of NADPH (McMahon and Nicholls, 1991). In addition, the total flavonoids of *Oldenlandia diffusa* could inhibit *N*-methyl-D-aspartate (NMDA) receptor-mediated currents in cultured rat hippocampal neurons (Cheng et al., 2006). Thus, whether quercetin affects miniature presynaptic glutamate release in glaucoma should be evaluated in glaucomatous RGCs.

The γ-aminobutyric acid (GABA) system is vital in local networks and interactions in different regions of the CNS, and it can be used as an alternative target to restore the balance between excitation and inhibition in the CNS (Rudolph and Mohler, 2014). Electrophysiological studies have shown that quercetin inhibits the expression of human homologous GABA1 receptor-mediated ionic currents in *Xenopus* oocytes (Goutman and Calvo, 2004) and antagonizes GABA type A (GABAA)-mediated responses and inhibits GABA type C (GABAC) receptors (Goutman et al., 2003). Thus, whether quercetin affects quantal presynaptic GABA release in glaucoma should be evaluated in glaucomatous RGCs.

Studying the mechanisms by which quercetin regulates the functions of the glutamate and GABA systems could provide important insights into the physiological and pathological roles of these systems in glaucoma. Therefore, electrophysiological electroretinography was used to detect changes in the photopic negative response (PhNR), which reflects RGC function. We conducted electrophysiological patch-clamp experiments in glaucomatous rat retinal slices to examine whether and how quercetin regulates glutamatergic and GABAergic synaptic transmission in the inner retina.

## Materials and Methods

### Ethics Statement

The animal procedures were approved by the Ethics Committee of Fudan University (No. 20160307-060) and were conducted in accordance with the Association for Research in Vision and Ophthalmology (ARVO) Statement for the Use of Animals in Ophthalmic and Vision Research. The researchers involved in the study made special efforts to reduce the number of animals used and to reduce their suffering.

### Animals

A total of 150 adult male Wistar rats aged over 2 months and weighing 180–220 g were used for all experiments (SLAC Laboratory Animal Co., Ltd., Shanghai, China). The rats were kept at 23 ± 2°C, 60–70% humidity and a 12 h light/dark cycle environment. Before the surgery, the rats were given intraperitoneal injections of ketamine (80 mg/kg) and xylazine (8 mg/kg) (volume ratio of 2:1) under deep anesthesia. Proparacaine hydrochloride (0.5% Alcaine; Alcon-Couvreur, Puurs, Belgium) was applied as a topical anesthetic, and 0.3% tobramycin (Tobres; Alcon-Couvreur) was applied to prevent postsurgical infection.

### Rat Model of Ocular Hypertension

As previously described (Mittag et al., 2000; Wu et al., 2010; Chen et al., 2011; Zhou et al., 2018), in the experiment of constructing the glaucoma model, the rats under deep anesthesia still showed relatively stable and normal respiratory rhythm. The left eye underwent a sham operation in which the veins were isolated without cauterization, while the episcleral veins located near the superior and inferior rectus muscles of the right eye were precisely isolated and cauterized. Additionally, 0.3% tobramycin (Tobres; Alcon-Couvreur) was applied to prevent postsurgical infection. After the surgery, morphine chloride (10 mg kg^-1^) was injected intraperitoneally to relieve the pain. Intraocular pressure (IOP) was measured every morning at nine o’clock using a calibrated tonometer (Tono-Pen XL; Mentor, Norwell, MA, United States) before surgery and at 1, 2, 3, and 4 weeks after surgery. IOP was recorded as the mean of five consecutive measurements with a deviation of <5% (Mittag et al., 2000).

### Measurement of the PhNR

Four weeks after the preparation of the chronic glaucoma rat model, an Espion Diagnosys System (Diagnosys LLC, Littleton, MA, United States) was used to record the whole-field electroretinogram. As mentioned before, the rats were anesthetized, and body temperature was maintained at 37°C using a thermal plate. After topical corneal anesthesia with Alcaine (Alcon), the PhNR was recorded with the Espion system with wire electrodes (PT-IR Tef., A=M system, Inc., Everett, WA, United States) placed on the corneal surface of the eye. The test eye was the site of the positive electrode; the ground electrode was inserted under the skin of the right leg, and the reference electrode was inserted under the skin of the nose. The electrical signals were recorded with two 3 mm tungsten rings precoated with 2.5% hydroxypropyl-methylcellulose solution (Gonak; Akorn, Lake Forest, IL, United States) attached to the corneal surfaces. At each intensity, 50 sweeps were recorded, with a sweep pretrigger time of 10 ms and a sweep posttrigger time of 150 ms. Light stimuli were delivered using a ColorDome unit at four different stimulus strengths (11.38 cd.s/m^2^-0.33 Hz, 11.38 cd.s/m^2^-1 Hz, 22.76 cd.s/m^2^-0.33 Hz, and 22.76 cd.s/m^2^-0.33 Hz) in a 4-step test. In each step, the stimulus frequency was 2 Hz, and a green light with an intensity of 10 cd/m^2^ was presented for 4 ms against a green background. The PhNR amplitude was defined as the peak of the negative wave following the b-wave that was measured relative to the baseline. Recording sessions lasted approximately 30 min, after which the animals were allowed to recover.

### Retinal Slice Preparation and Electrophysiological Recordings

The rat eyeballs were removed quickly and placed in ice-cold oxygenated (95% O_2_/5% CO_2_) cutting solution for 30 min: 26 mM NaHCO_3_, 1.25 mM NaH_2_PO_4_, 124 mM sucrose, 3 mM KCl, 3 mM sodium pyruvate, 0.2 mM CaCl_2_, 3.8 mM MgCl_2_, and 10 mM glucose (pH 7.4). Then, the retinas were buckled back on the filter paper to ensure that the RGC layer was close to the filter paper. The retina was then cut into 200-μm retinal slices using a manual slicer, and these slices were then incubated in oxygen-saturated artificial cerebrospinal fluid [ACSF (in mM): 125 NaCl, 3 KCl, 1.25 NaH_2_PO_4_, 15 D-glucose, 1 MgCl_2_, 2 CaCl_2_, and 26 NaHCO_3_ (pH 7.35–7.45)] for approximately 40 min. In 150 adult male Wistar rats, six or seven retinal slices were evaluated per retina, and the number of cells (n) for each comparison is given in the corresponding results.

Using an infrared differential interference contrast (IR-DIC)-sensitive charge-coupled device (CCD) camera (Nikon, Tokyo, Japan) system and a water-immersion objective lens at 40× magnification, RGCs were selected for whole-cell patch-clamp recording according to their position, morphology, Lucifer yellow staining (Li et al., 2016). The holding potential was set at -70 mV. An Axopatch MultiClamp 700B amplifier (Axon Instruments, Foster City, CA, United States; sampling frequency = 10 kHz, filter frequency = 1 kHz) coupled to a Digidata 1440A digital analog converter system (Axon Instruments, Foster City, CA, United States) and a personal computer running Clampex and Clampfit 10.2 software (version 10, Axon Instruments, Molecular Devices LLC., Sunnyvale, CA, United States) were used to collect the recordings. For recordings of inhibitory postsynaptic currents (IPSCs), the holding potential was clamped at -70 mV using an Axopatch MultiClamp 700B amplifier. The frequency and amplitude parameters of miniature synaptic currents were analyzed by MiniAnalysis (version 4.3.1; Synaptosoft Inc., Fort Lee, NJ, United States), with the minimum acceptable amplitude set at 5 pA. The membrane current signal was low-pass filtered at 5 Hz with the eight-pole Bessel filter. In each RGC, analysis was performed at 5 min of control recording, at the time of agonist (quercetin) application, and after fresh extracellular rinse was resumed. The frequency of the action potential burst and the changes of membrane potential before and after drug use were compared with Clampfit 10.2 software (Molecular Devices LLC., Sunnyvale, CA, United States).

The patch pipettes had open-tip resistances of 4–8 MΩ when filled with an intracellular solution containing (in mM) 150 CsCl, 10 HEPES, 1 MgCl_2_, 1 EGTA, 0.1 CaCl_2_, 4 Mg-ATP, and 0.4 Na-GTP (pH 7.2). Excitatory postsynaptic currents (EPSCs) were recorded in the whole-cell configuration in voltage clamp mode with 3–5-MΩ electrodes filled with a solution containing (in mM) 120 CsMeSO_3_, 5 NaCl, 0.2 GTP-Na, 2 ATP-Mg, 10 TEA-Cl, 2 EGTA, and 10 HEPES (pH 7.2; adjusted with CsOH). QX314 (lidocaine N-ethyl bromide; 2.0 mM) was added to the pipette solution to block rapid Na^+^ currents. Action potentials were recorded in the whole-cell configuration in current clamp mode with a solution containing (in mM) 120 potassium D-gluconate, 10 phosphocreatine, 0.3 GTP-Na, 4 ATP-Mg, 1 EGTA, 10 HEPES, 1 MgCl_2_, and 0.1 CaCl_2_ (pH 7.2; adjusted with KOH).

### Drug Administration

The tip of a needle was inserted into the superior hemisphere of the eye at a 45° angle through the sclera into the vitreous body. Some rats received an intravitreal injection of 2 μL of quercetin 10 μM. The control eyes received intravitreal injections of 2 μL PBS, and this protocol was repeated weekly thereafter. The following drugs were applied using a gravity-fed superfusion system for patch-clamp recordings: 2-(3,4-dihydroxyphenyl)-3,5,7-trihydroxy-4H-chromen-4-one (quercetin, 10 μM), tetrodotoxin (TTX, 1 μM, to abolish spontaneous action potentials), 6-cyano-7-nitroquinoxaline-2,3-dione (CNQX, 10 μM) and D-2-amino-5-phosphonovalerate (AP5, 50 μM) (to block ionotropic glutamate receptors), SR95531 [2-(3-carboxypropyl)-3-amino-6-methoxyphenyl-pyridazinium bromide, 10 μM, to block GABAARs] and strychnine (5 μM, to block glycine receptors). All drugs were purchased from Sigma-Aldrich.

### Data Analysis

Data are expressed as the mean ± SEM. The mean frequency and amplitude before and after drug administration were statistically analyzed by a two-sample *t*-test. The data reported were analyzed by one-way ANOVA with Bonferroni *post hoc* tests for multiple comparisons. The analysis was completed using SPSS software (17.0; SPSS Inc., Chicago, IL, United States). *P* < 0.05 was considered to represent a significant difference.

## Results

### Quercetin Can Reduce Functional Impairment Induced by Chronic Glaucoma

**FIGURE 1:**
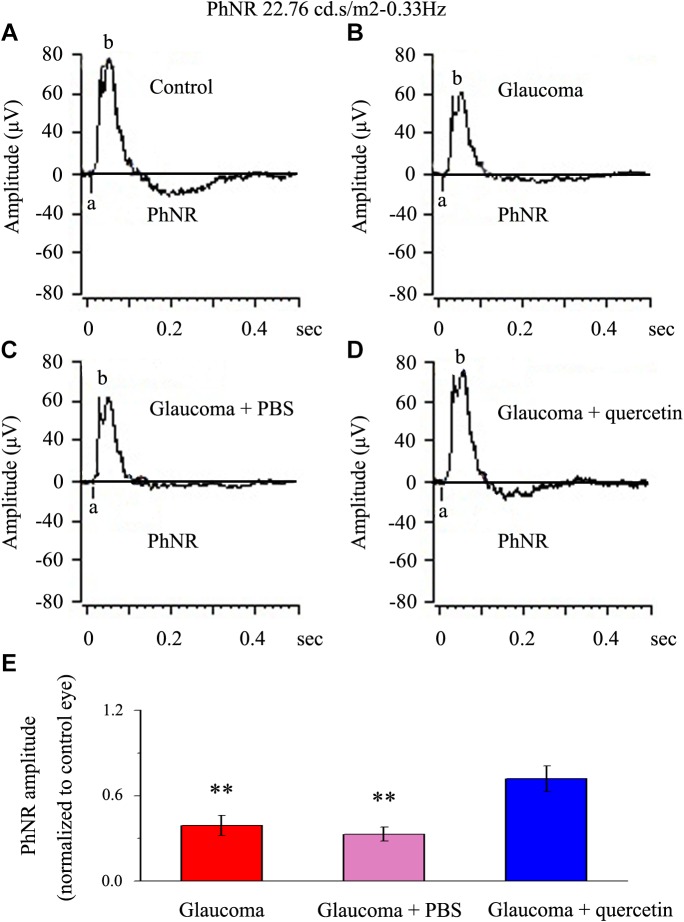
PhNR of normal and glaucomatous rat eyes. **(A,B)** Representative traces of the “a” wave, the “b” wave, and PhNR in a control eye **(A)** and a glaucomatous eye **(B)** in step 3 with the stimulus applied at 22.76 cd.s/m^2^–0.33 Hz. **(C,D)** Representative waves in a PBS-treated glaucomatous eye **(C)** and quercetin-treated glaucomatous eye **(D)** in the same step and with the same stimulus used in **(A,B)**. **(E)** Quantitative analysis of PhNR amplitude (*n* = 12). The amplitude was normalized to the amplitude in control retinas. ^∗∗^*p* < 0.01 (one-way analysis of variance). a, “a” wave; b, “b” wave; PhNR, photopic negative response.

We first assessed whether quercetin could improve retinal function impairment induced by glaucoma; for this purpose, we used the a functional electroretinography experiment. We analyzed the photopic negative response (PhNR) reflecting the function of the inner retina. The PhNR amplitude was positively correlated with the functional status of retinal ganglion cells (RGCs) (Niyadurupola et al., 2013; Preiser et al., 2013; Liu et al., 2014). Figure 1 presents data from the rats 4 weeks after chronic glaucoma modeling, which indicated that the PhNR amplitude was significantly decreased in the glaucoma group without intravitreal PBS injection (39% ± 7%, *p* < 0.01, *n* = 12, Figure 1A,B,E) and the glaucoma group with intravitreal PBS injection (33% ± 5%, *p* < 0.01, *n* = 12, Figure 1A,C,E) compared with the control group. Quercetin was injected into the vitreous cavity once a week (four times in total), and we found that the PhNR amplitude increased to 72% ± 9% of the control group (*p* > 0.05, *n* = 12, Figure 1A,D,E).

### Baseline Miniature Postsynaptic Currents (mPSCs) Were More Common in OFF-Type RGCs Than in ON-Type RGCs

**FIGURE 2:**
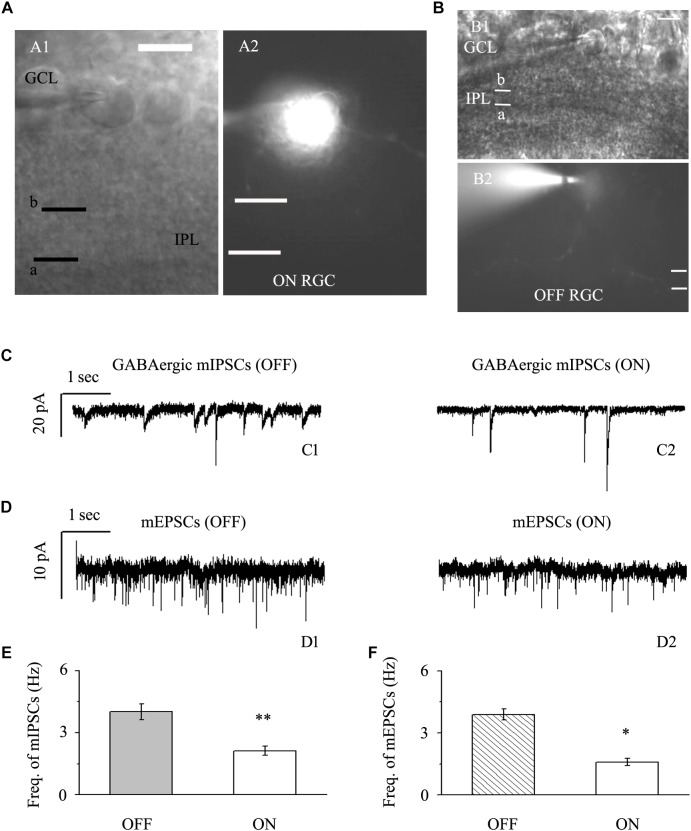
Baseline sIPSCs and sEPSCs were more common in OFF-type RGCs than in ON-type RGCs. **(A,B)** The infrared interference phase microscope image in **(A)** shows all of the retinal layers. Representative Lucifer Yellow-filled OFF-type RGCs with dendritic arborizations in the distal (b) parts of the IPL are shown (A1, A2). Representative Lucifer Yellow-filled OFF-type RGCs with dendritic arborizations in the proximal (a) and distal (b) parts of the IPL are also shown (B1, B2). GCL, ganglion cell layer; IPL, inner plexiform layer. Scale bar, 5 μm. **(C)** Representative traces showing the frequency of GABAergic mIPSCs in both OFF-type RGCs (C1) and ON-type RGCs (C2). Vertical scale bar, 20 pA; horizontal scale bar, 1 s. **(D)** Representative traces showing the frequency of glutamatergic sEPSCs in both OFF-type RGCs (D1) and ON-type RGCs (D2). Vertical scale bar, 10 pA; horizontal scale bar, 1 s. **(E)** Summarized data from 14 RGCs showing that baseline mIPSCs were more common in OFF-type RGCs than in ON-type RGCs. **(F)** Summarized data from 12 RGCs showing that baseline mEPSCs were more common in OFF-type RGCs than in ON-type RGCs. ^∗^*p* < 0.05 and ^∗∗^*p* < 0.01, unpaired Student’s *t*-test.

We focused on two types of RGCs defined by their location and the shape of their protuberances (Famiglietti, and Kolb, 1976; Margolis and Detwiler, 2007). In this study, the effects of quercetin on afferent GABAergic and glutamatergic synaptic terminals were detected mainly in OFF-type (*n* = 42) and ON-type (*n* = 17) RGCs. Based on established morphological and physiological indicators, OFF-type RGCs (71%) and ON-type RGCs (29%) were identified (Famiglietti, and Kolb, 1976; Margolis and Detwiler, 2007). Dendritic terminals were characterized morphologically according to the locations of the OFF- and ON-type RGCs and based on whether the dendrites terminated in the proximal (a) or distal (b) parts of the inner plexiform layer (IPL) (Figure 2A,B). The baseline frequency of GABAergic mIPSCs in OFF-type RGCs was higher than that in ON-type RGCs (4.01 ± 0.38 Hz vs. 2.12 ± 0.22 Hz, *n* = 14, *p* < 0.01; Figure 2C,E). The results were similar for glutamatergic mEPSCs: mEPSCs were more abundant in OFF-type RGCs than in ON-type RGCs (3.89 ± 0.27 Hz vs. 1.59 ± 0.18 Hz, *n* = 12, *p* < 0.05; Figure 2D,F).

### Chronic Glaucoma Altered mIPSC but Not mEPSC Frequency in Both the OFF- and ON-Type RGCs

The retina contains a complete neural network structure. In the inner retina of mammals, RGCs receive glutamatergic excitation from presynaptic bipolar cells or GABA or glycine input from amacrine cells; bipolar cells, amacrine cells and RGCs constitute a neural circuit. This circuit receives and processes visual information through the regulation of synaptic transmission. Synaptic dysfunction is the direct cause of many types of retinopathy. Moreover, Li et al. found that there were changes in the structure and function of dendritic synapses in diseases of the central nervous system. In cerebral ischemia, if the blood flow is seriously decreased, the number of dendritic spines drops sharply within 10 min, and the morphology and structure also change, affecting the synaptic activity. If blood flow is restored within 1 h, the morphology and function of dendrites and dendritic spines is restored to some extent. Thus, the reversibility and importance of synaptic regulation may be important determinants of the optimal window of time for drug therapy.

**FIGURE 3:**
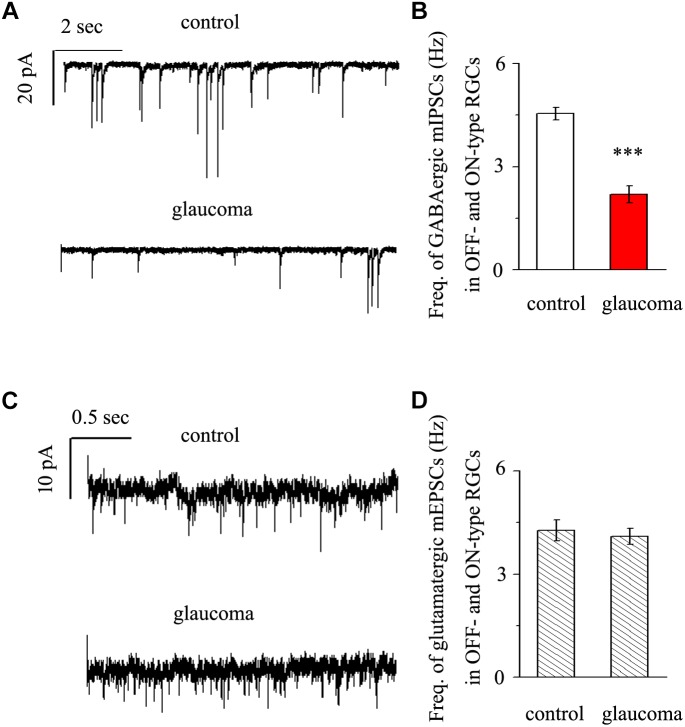
Effects of chronic glaucoma on retinal mIPSCs and mEPSCs. **(A)** Representative traces of voltage clamp recordings of GABAergic mIPSCs in the presence of TTX (1 μM) in control and glaucomatous retinas. The baseline frequency of mIPSCs was markedly lower in glaucomatous retinas than in control retinas. Vertical scale bar, 20 pA; horizontal scale bar, 2 s. **(B)** Bar graphs showing the mean frequency of mIPSCs in control and glaucomatous retinas. **(C)** Representative traces of voltage-clamp recordings of glutamatergic mEPSCs in the presence of TTX (1 μM) in control and glaucomatous retinas. The baseline frequency of mEPSCs did not differ between glaucomatous retinas and control retinas. Vertical scale bar, 10 pA; horizontal scale bar, 0.5 s. **(D)** Bar graphs showing the mean frequency of mEPSCs in control and glaucomatous retinas. ^∗∗∗^*p* < 0.001, unpaired Student’s *t*-test.

We next performed whole-cell patch-clamp studies to examine whether the kinetics of mIPSCs and mEPSCs in both the OFF- and ON-type RGCs were altered in glaucomatous retinas. mIPSCs were recorded using electrodes filled with CsCl solution (electrode impedance 4–8 MΩ) containing TTX (1 μM), CNQX (10 μM), AP5 (50 μM) and strychnine (5 μM). Individual RGCs were clamped at a holding potential of -70 mV. Since both types of RGC presented the same effect, we combined the responses of the two types of RGC to present these results statistically. Figure 3A shows traces of GABAergic mIPSCs from RGCs in control and glaucomatous retinal slices. The mIPSC frequency was 4.54 ± 0.18 Hz in control RGCs and 2.19 ± 0.25 Hz in glaucomatous RGCs (*n* = 11, *p* < 0.001; Figure 3B). However, the mean mIPSC amplitude was not significantly different between control and glaucomatous RGCs. mEPSCs were recorded using electrodes filled with a solution containing TTX (1 μM), SR95531 (10 μM) and strychnine (5 μM). Individual RGCs were clamped at a holding potential of -70 mV. The mEPSC frequency did not differ significantly between control and glaucomatous RGCs (4.27 ± 0.31 Hz vs. 4.09 ± 0.23 Hz, *n* = 9, *p* > 0.05; Figure 3C,D).

### Quercetin Increased Both the Frequency and Amplitude of Miniature IPSCs in OFF-Type Glaucomatous RGCs

**FIGURE 4:**
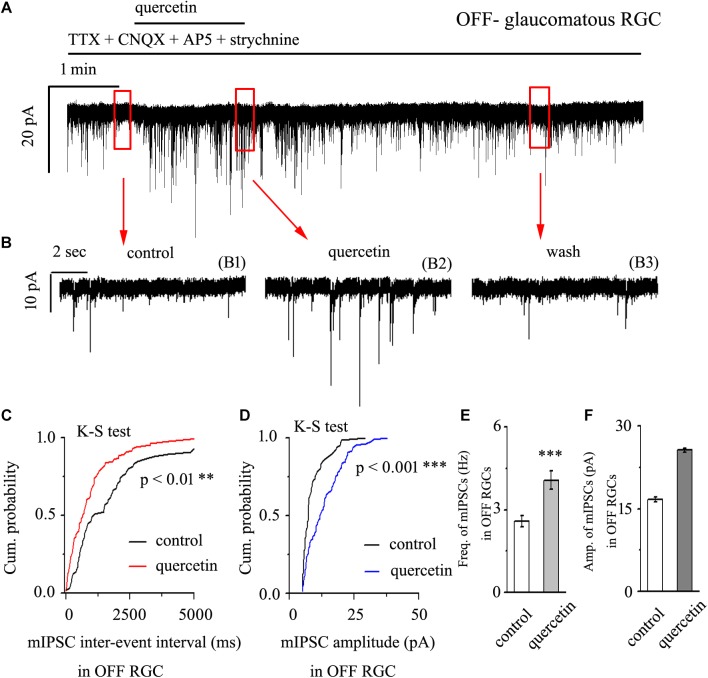
Quercetin increased the frequency and amplitude of GABAergic mIPSCs in OFF-type RGCs. **(A)** Representative traces showing the effect of 10 μM quercetin on mIPSCs. Vertical scale bar, 20 pA; horizontal scale bar, 1 min. **(B)** Recordings from a large-scale representative experiment under control conditions (B1), during quercetin treatment (B2), and during recovery (B3). **(C,D)** Cumulative frequency and amplitude distributions of GABAergic mIPSCs in a representative neuron under control conditions and during quercetin treatment showing that quercetin caused the cumulative frequency curve to shift significantly to the left and the cumulative amplitude curve to shift significantly to the right. **(E,F)** Summarized data on the frequency **(E)** and amplitude **(F)** of GABAergic mIPSCs (*n* = 9; from six slices). ^∗∗^*p* < 0.01 and ^∗∗∗^*p* < 0.001 compared with the control conditions; paired Student’s *t*-test.

After preincubation with 1 μM TTX, treatment with 10 μM quercetin significantly increased both the frequency and amplitude of mIPSCs in OFF-type glaucomatous RGCs (Figure 4A,B). The effects of quercetin on the cumulative distributions of the frequency and amplitude of GABAergic mIPSCs, as assessed by the Kolmogorov–Smirnov test, are shown in Figure 4C (*n* = 10, *p* < 0.01) and Figure 4D (*n* = 10, *p* < 0.001), respectively. The cumulative frequency curve shifted significantly to the left after quercetin administration, while the cumulative amplitude curve shifted significantly to the right. The mIPSC frequency increased from 2.59 ± 0.2 Hz to 4.08 ± 0.33 Hz (*p* < 0.001; *n* = 9; paired Student’s *t*-test, Figure 4E). The peak amplitude increased from 16.75 ± 0.44 pA to 25.68 ± 0.38 pA (*p* < 0.01; *n* = 9; paired Student’s *t*-test, Figure 4F). The quercetin-induced responses started within 5 min and were reversible. At the end of the experiments, application of SR95531 (10 μM) abolished all of the mIPSCs.

### Quercetin Significantly Increased the Frequency, but Not the Amplitude, of GABAergic mIPSCs in ON-Type RGCs

**FIGURE 5:**
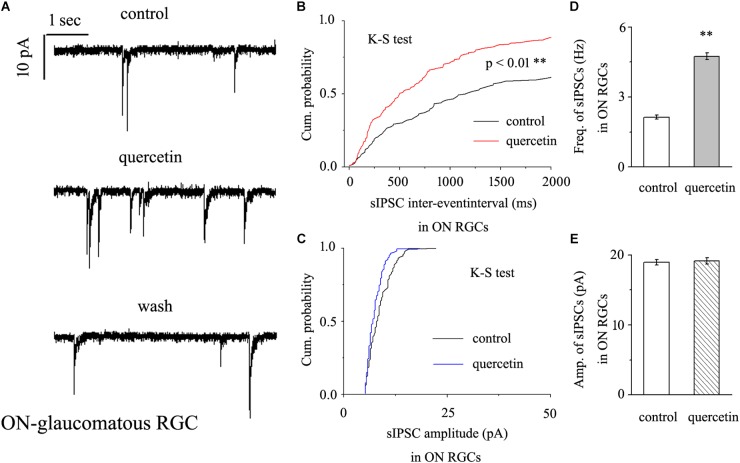
Quercetin significantly increased the frequency, but not the amplitude, of mIPSCs in ON-type RGCs. **(A)** Representative recordings showing that quercetin increased the frequency but not amplitude of mIPSCs. (**A**, top) control condition; (**A**, middle) during quercetin treatment; (**A**, bottom) recovery period. Vertical bar, 10 pA; horizontal bar, 1 s. **(B,C)** Cumulative interevent interval and amplitude distributions of mIPSCs in a representative neuron during a control recording and after quercetin application. Quercetin significantly shifted the distribution of interevent intervals to the left **(B)** but did not shift the distribution of mIPSC amplitudes **(C)**. The quercetin-induced changes in the distribution of interevent intervals were statistically significant (*n* = 11). ^∗∗^*p* < 0.01, Kolmogorov–Smirnov test. **(D,E)** Summarized data for the frequency **(D)** and amplitude **(E)** of mIPSCs (frequency: *n* = 13; amplitude: *n* = 9). ^∗∗^*p* < 0.01, paired Student’s *t*-test.

In further assessments of the effect of quercetin on spontaneous IPSCs (sIPSCs) in ON-type RGCs, quercetin (10 μM) significantly increased the frequency, but not the amplitude, of sIPSCs (Figure 5A). The effects of quercetin on the cumulative distributions of the frequency and amplitude of sIPSCs, as assessed by the Kolmogorov–Smirnov test, are shown in Figure 5B (*n* = 11, *p* < 0.01) and Figure 5C (*n* = 11, *p* > 0.05), respectively. The cumulative frequency curve shifted significantly to the left. The cumulative amplitude curve did not differ between before and after quercetin administration. The frequency increased from 2.89 ± 0.15 Hz to 4.98 ± 0.31 Hz (*n* = 8, *p* < 0.01; Figure 5D), while the amplitude did not change (18.15 ± 0.62 pA in the control group vs. 19.12 ± 0.75 pA in the treated group; *n* = 8, *p* > 0.05; Figure 5E). These effects took place within 6–8 min, reached a maximum in 10–15 min, and lasted for 20–30 min before full recovery. At the end of the experiments, 10 μM SR95531 blocked all of the GABAergic mIPSCs.

### Quercetin Significantly Inhibited the Frequency, but Not the Amplitude, of Glutamatergic mEPSCs in OFF-Type RGCs

**FIGURE 6:**
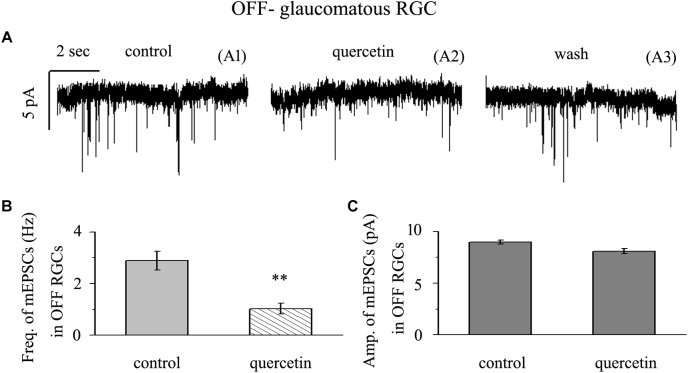
Quercetin significantly inhibited the frequency, but not the amplitude, of mEPSCs in OFF-type RGCs. **(A)** Representative traces showing the effect of 10 μM quercetin on glutamatergic mEPSCs in OFF-type RGCs. (A1) Control conditions; (A2) during quercetin treatment; (A3) recovery. Vertical bar, 5 pA; horizontal bar, 2 s. **(B)** Summarized data from 14 OFF-type RGCs showing that 10 μM quercetin significantly decreased the average frequency of glutamatergic mEPSCs. **(C)** Summarized data from 14 OFF-type RGCs showing that 10 μM quercetin did not change the average amplitude of glutamatergic mEPSCs. ^∗∗^*p* < 0.01, paired Student’s *t*-test.

Application of quercetin (10 μM) significantly decreased the frequency of mEPSCs (Figure 6A) in OFF-type RGCs from a baseline of 2.89 ± 0.36 Hz to 1.03 ± 0.21 Hz (*n* = 14, *p* < 0.01; Figure 6B). The amplitudes of the mEPSCs before and during quercetin application were 8.98 ± 0.18 pA and 8.11 ± 0.27 pA, respectively (*n* = 14, *p* > 0.05; Figure 6C). These effects took place within 5 min, reached a maximum in 10–15 min, and lasted for 20–30 min before full recovery. At the end of the experiments, 10 μM CNQX and 50 μM AP5 blocked all of the mEPSCs.

### Quercetin Significantly Inhibited the Frequency, but Not the Amplitude, of Glutamatergic mEPSCs in ON-Type RGCs

**FIGURE 7:**
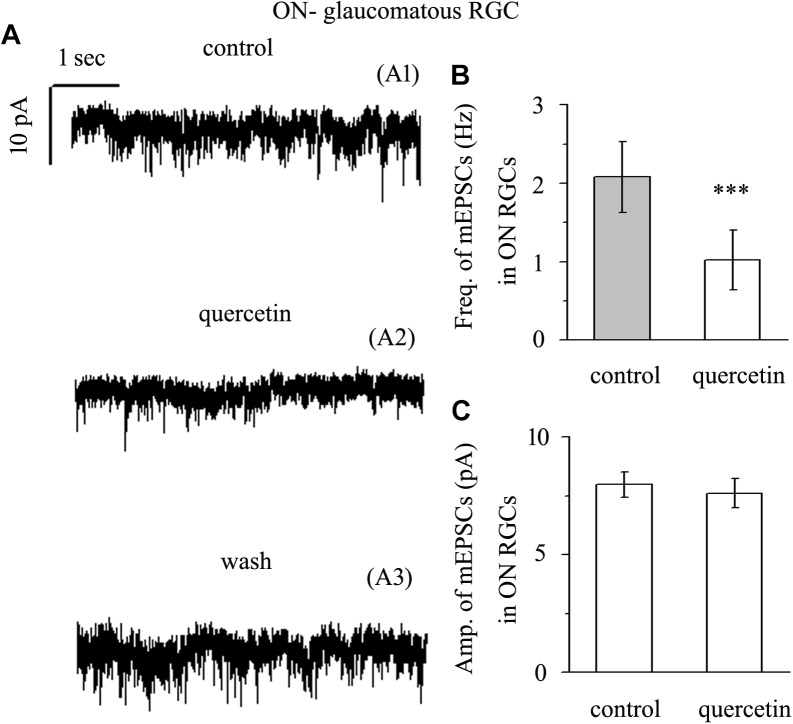
Quercetin significantly inhibited the frequency, but not the amplitude, of mEPSCs in ON-type RGCs. **(A)** Representative traces showing the effect of 10 μM quercetin on glutamatergic mEPSCs in ON-type RGCs. (A1) Control conditions; (A2) during quercetin treatment; (A3) recovery. Vertical bar, 10 pA; horizontal bar, 1 s. **(B)** Summarized data from 10 ON-type RGCs showing that 10 μM quercetin significantly decreased the average frequency of glutamatergic mEPSCs. **(C)** Summarized data from 10 ON-type RGCs showing that 10 μM quercetin did not change the average amplitude of glutamatergic mEPSCs. ^∗∗∗^*p* < 0.001, paired Student’s *t*-test.

Application of quercetin (10 μM) also significantly decreased the frequency of mEPSCs (Figure 7A) in ON-type RGCs from a baseline of 2.08 ± 0.45 Hz to 1.02 ± 0.38 Hz (*n* = 10, *p* < 0.001; Figure 7B). The amplitudes of the mEPSCs before and during quercetin application were 7.98 ± 0.55 pA and 7.62 ± 0.62 pA, respectively (*n* = 10, *p* > 0.05; Figure 7C). These effects took place within 5 min, reached a maximum in 10–15 min, and lasted for 20–30 min before full recovery. At the end of the experiments, 10 μM CNQX and 50 μM AP5 blocked all of the mEPSCs.

### Quercetin Modulated the Spontaneous Firing and Membrane Potential of RGCs

**FIGURE 8:**
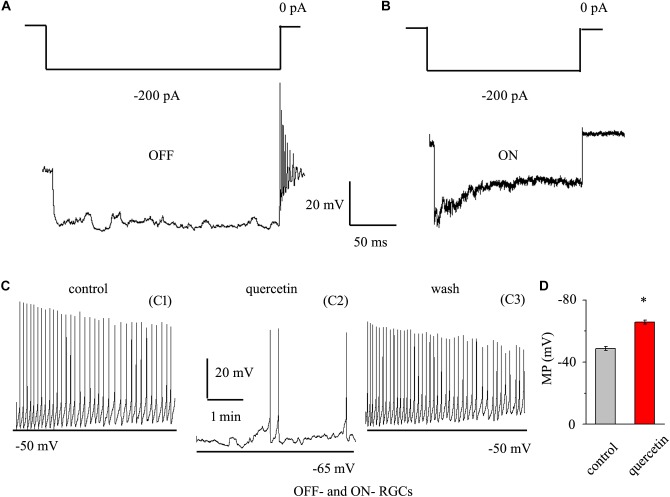
Quercetin hyperpolarized RGCs and decreased the firing rate. **(A,B)** Top trace: responses to a –200 pA current step in OFF-type RGCs **(A)** and ON-type RGCs **(B)**. Negative current injection led to rebound burst firing in OFF-type RGCs but not in ON-type RGCs. Vertical bar, 20 mV; horizontal bar, 50 ms. **(C)** Current clamp recording of a representative RGC. Note that quercetin caused hyperpolarization and decreased the firing rate. Recordings under control conditions (C1), during application of quercetin (C2), and during washout (C3) are shown on an expanded time scale. **(D)** Bar graph summarizing the changes in MP. Quercetin can hyperpolarize MP. MP, membrane potential. ^∗^*p* < 0.05, paired Student’s *t*-test.

As quercetin enhanced the inhibitory inputs and decreased the excitatory inputs of RGCs, we sought to determine the overall effect of quercetin on RGC activity. RGCs display spontaneous firing under physiological conditions in rats (Li et al., 2017) and mice (Margolis and Detwiler, 2007). The electrophysiological characteristics of the RGCs were confirmed by determining the responses of the RGCs to a negative current step in current clamp mode (Margolis and Detwiler, 2007; Zhang et al., 2011). Negative current (-200 pA) injection led to rebound burst firing in OFF-type RGCs but not in ON-type RGCs (Figure 8A,B). We compared the spontaneous firing rate and membrane potential between OFF- and ON-type RGCs and found no significant differences between the types [frequency: 1.86 ± 0.52 Hz for OFF-type RGCs (*n* = 15) and 1.65 ± 0.32 Hz for ON-type RGCs (*n* = 12), *p* > 0.05; membrane potential: -48.7 ± 1.2 mV for OFF-type RGCs (*n* = 15) and -49.5 ± 1.1 mV for ON-type RGCs (*n* = 12), *p* > 0.05]. Therefore, the data from both types were pooled for this experiment. We further investigated whether quercetin modulates the spontaneous firing and membrane potential of RGCs in current clamp mode. As shown in Figure 8C,D, the perfusion of quercetin significantly decreased the number of spontaneous action potentials, but the firing rate gradually returned to control levels with washout of quercetin. On average, the firing rate decreased to 16.9 ± 8% of the control rate after 3 min of quercetin perfusion (*n* = 9, *p* < 0.01). Data analysis showed that the cellular membrane potential hyperpolarized to -65.8 ± 1.4 mV from a control value of -48.8 ± 1.3 mV upon quercetin treatment (*n* = 9, p < 0.05; Figure 8D). These results demonstrate that the overall effect of quercetin on RGCs is inhibitory.

## Discussion

In the present study, we first found that quercetin can reverse the decline in PhNR, a functional indicator of RGC, caused by chronic glaucoma. Second, we determined that the baseline frequencies of mIPSCs and mEPSCs in OFF-type RGCs were greater than those in ON-type RGCs. Third, we revealed differences in the baseline frequencies, but not the amplitudes, of GABAergic mIPSCs in RGCs between control and glaucomatous retinal slices. Fourth, we found that the frequency and amplitude of glutamate receptor-mediated glutamatergic mEPSCs were not significantly different between control and glaucomatous RGCs. Finally, we revealed that quercetin increased miniature GABAergic (inhibitory) neurotransmission to RGCs and decreased miniature glutamatergic (excitatory) neurotransmission, thus alleviating the excitability of RGCs in glaucomatous retinal slices.

We evaluated the frequencies and amplitudes of mIPSCs and mEPSCs in ON- and OFF-type RGCs and found significant differences in the frequencies, but not the amplitudes, of mIPSCs and mEPSCs between these subtypes. The frequencies of both mIPSCs and mEPSCs were greater in OFF-type RGCs than in ON-type RGCs. Previous studies have reported that compared with the ON-type RGCs, the OFF-type RGCs present a higher peak velocity, and it is easy for depolarization to induce an action potential and produce a greater peak in the current injection (Myhr et al., 2001). In addition, OFF-type RGCs exhibit more rapid declines in both structural and functional organization than ON-type RGCs (Della Santina et al., 2013). What are the causes of these differences in mIPSCs and mEPSCs between ON- and OFF- type RGCs? To some extent, the excitability and electrical conductance of OFF-type RGCs are greater than those of ON-type RGCs. The larger mIPSCs and mEPSCs may simply reflect larger GABAergic and glutamatergic mediated synaptic inputs in OFF-type cells. In the chronic glaucoma animal model, dendrites stratifying in the OFF sublamina are the first to undergo structural and functional changes (Della Santina et al., 2013; El-Danaf and Huberman, 2015). Why RGCs located in the OFF sublamina are more susceptible to increased intraocular pressure. El-Danaf and Huberman believed that the proximity of this sublamina to the vasculature made it particularly vulnerable to vascular damage (El-Danaf and Huberman, 2015). Because of the difference in OFF and ON structure and function, we need to study specific RGC types from the perspective of sequence and temporal relationships. This allows us to validate and design neuroprotective strategies from a functional specificity perspective. However, we did not find that the two types of RGC had different effects on quercetin. We found that quercetin affects synaptic transmission to both ON- and OFF- type RGCs.

Previous studies have reported the regulation of quercetin on GABA receptors, mainly focusing on exogenous GABA delivery, thus acting on the corresponding postsynaptic receptors. Quercetin reduces spontaneous and electrically evoked GABAergic IPSCs in prefrontal cortical slices and concentration-dependently reduces GABA-induced currents in cultured cortical neurons (Fan et al., 2018). However, under our experimental conditions, the response of GABA release to quercetin was different from that described in previous studies. Moreover, quercetin-mediated GABA release was not inhibited by pretreatment with TTX, indicating that the observed effects were not dependent on the activity of Na^+^ channels. The association of quercetin with the increased frequency of mIPSCs may indicate an increased probability of terminal inhibitory neurotransmitter release or increased excitability of intermediate neuron axons. Additional studies examining GABA-induced currents and GABA receptor activity are needed to confirm whether postsynaptic effects are involved in the observed responses of RGCs to quercetin.

It has also been reported that quercetin can reduce the current induced by NMDA, but the effect on the AMPA receptor current is weak (Goutman et al., 2003). In our study, we studied how quercetin affected the frequency and amplitude of mEPSCs. This strong reduction in frequency also suggests a reduction in presynaptic effects or network excitability. In fact, it has been reported that some quercetin can open calcium-activated potassium channels (Koh et al., 1994), which can reduce the release of synaptic vesicles. Our study provides evidence that quercetin reduces glutamate release from nerve terminals. Excessive release of glutamate is considered a key factor in the neuropathology of chronic neurodegenerative diseases (Meldrum, 2000). Therefore, it is reasonable to believe that decreased glutamate release from nerve terminals is an important protective mechanism of quercetin against excitatory neurotoxicity. Given all of our findings, we propose that quercetin stimulation and the resulting inhibition of glutamate release by RGCs constitute an effective neuroprotective strategy for the treatment of glaucoma. Our findings also contribute to the current understanding of the synaptic effects and mechanisms of RGC injury.

The present data help to identify the structural determinants and mechanisms by which quercetin regulates neurotransmitter systems. Thus, we hope that our work will ultimately contribute to the development of selective drugs targeting glutamatergic and GABAergic neuronal subtypes.

## Data Availability

All datasets generated for this study are included in the manuscript and/or the supplementary files.

## Ethics Statement

Ethical approval for the animal procedures were approved by the Ethics Committee of Fudan University (No. 20160307-060) and were conducted in accordance with the Association for Research in Vision and Ophthalmology (ARVO) Statement for the Use of Animals in Ophthalmic and Vision Research. The researchers involved in the study made special efforts to reduce the number of animals used and to reduce their suffering.

## Author Contributions

XZ and JW designed the research. XZ and GL conducted the research. BY analyzed the data. XZ wrote the manuscript. JW modified the manuscript.

## Conflict of Interest Statement

The authors declare that the research was conducted in the absence of any commercial or financial relationships that could be construed as a potential conflict of interest.
